# Integrated study of hydrochemistry, quality and risk to human health of groundwater in the upper reaches of the Wulong River Basin

**DOI:** 10.1371/journal.pone.0312000

**Published:** 2024-10-21

**Authors:** Chunwei Liu, Caiping Hu, Xiancang Wu, Changsuo Li, Xuan Wu, Chuanlei Li, Bin Sun, Huan Qi, Qingyu Xu

**Affiliations:** 1 Shandong Provincial Geo-mineral Engineering Exploration Institute (No. 801 Hydrogeological and Engineering Geology Brigade of Shandong Provincial Bureau of Geology and Mineral Resources), Jinan, China; 2 Shandong Engineering Research Center for Environmental Protection and Remediation on Groundwater (Under Preparation), Jinan, China; 3 Key Laboratory of Groundwater Resources and Environment, Shandong Provincial Bureau of Geology & Mineral Resources, Jinan, China; 4 School of Water Conservancy and Environment, University of Jinan, Jinan, China; National College Autonomous, INDIA

## Abstract

Groundwater, a vital source of water supply, is currently experiencing a pollution crisis that poses a significant risk to human health. To understand the hydrochemical formation mechanisms, quality and risk to human health of groundwater in the upper reaches of the Wulong River basin, 63 sets of groundwater samples were collected and analyzed. A combination of mathematical statistics, correlation analysis, Gibbs diagram, ion ratio, and cation exchange were comprehensively employed for hydrochemical analysis, and further water quality index (WQI) and human health risk assessment were conducted. The results indicate that groundwater is generally neutral to weakly alkaline. The dominant cations in the groundwater are Ca^2+^ and Mg^2+^, while the main anions are HCO_3_^−^ and SO_4_^2−^. The hydrochemical types of groundwater mainly include HCO_3_·SO_4_-Ca, HCO_3_-Ca and HCO_3_-Na. The diverse hydrochemical types are mainly due to the fractured and discontinuous nature of the aquifers. The hydrochemical characteristics are influenced by the dissolution of silicate and carbonate minerals, cation exchange processes, and anthropogenic pollution. The presence of NO_3_^−^ in groundwater is primarily attributed to agricultural activities. The groundwater is mainly categorized as "Good" (36.6%) and "Poor" (60.8%). "Very poor" and "Excellent" categories are rare, accounting for only 1.2% and 1.4%, respectively, and no samples are classified as "Non-drinkable". The *Ew*_*i*_ for NO_3_^−^ is the highest, indicating severe contamination by anthropogenic NO_3_^−^ pollution. Human health risk assessment reveals that water samples posing exposure risks account for 82.54% for children and 79.37% for adults. This study highlighted that anthropogenic nitrate pollution has deteriorated groundwater quality, posing risks to human health. It also suggests an urgent need to enhance research and protective measures for groundwater in similar regions, such as the Shandong Peninsula.

## 1. Introduction

Groundwater, as a vital natural resource for fulfilling the daily needs of humanity, plays a crucial role in maintaining the balance of the evolution of ecological systems [1–3]. It significantly contributes to ensuring the livelihoods of urban and rural residents and supports sustainable economic and social development [4]. Ongoing economic and social development, coupled with continuous population growth, has resulted in improper development, utilization, and pollution from human production and lifestyle, leading to the severe degradation of groundwater resources [5]. Attention to groundwater has extended beyond groundwater quantity concerns to groundwater quality as water quality has implications for water safety and human health, and research into groundwater quality can provide useful information for sustainable management of water resources within socioeconomic development [6, 7].

The hydrochemical composition of groundwater is a key determinant of its quality, influenced naturally by the chemical composition of its recharge sources, typically atmospheric precipitation, water-rock interactions, and the mixing of different water [4, 8]. In river basins, pollution from rivers and lakes can infiltrate and contaminate groundwater, heightening the risk of pollution [3, 9, 10]. With increasing human activities, pollutants generated by these activities are further contaminating groundwater, making the study of groundwater hydrochemistry more complex and challenging [5]. Therefore, understanding the impact of human activities on the hydrochemical characteristics and formation of groundwater is essential for the rational development, utilization, and protection of this vital resource [11]. Numerous studies have identified issues such as nitrogen pollution and increased chloride levels in groundwater, both of which are attributable to human activities [4, 12, 13]. The prevailing trend in current groundwater hydrochemical research involves the combined use of various methods to characterize water chemistry and the controlling factors such as Gibbs diagrams, Piper diagrams, the ion ratio and statistical analyses [12, 14, 15]. Wu et al [4] studied the formation mechanisms of groundwater in Jinan, employing methods such as Piper diagrams, ion ratios, correlation analysis, and factor analysis indicating the impact of human activities on groundwater hydrochemistry.

Recent researches have concentrated on assessing groundwater pollution and quality, as both are directly linked to human health [16, 17]. Poor groundwater quality poses significant health risks to human, highlighting the necessity of using the Water Quality Index (WQI) globally to evaluate overall groundwater suitability for drinking [18–20]. The WQI serves as an effective tool for characterizing water quality, condensing extensive water quality data into a single comprehensible number that represents a regional groundwater quality index [6, 15]. Ravindra et al [12] assessed groundwater quality in Guntur district, Andhra Pradesh, India, using the WQI, finding that 75% of samples were of poor quality and 25% were of very poor quality, covering 85.84% and 14.06% of the study area respectively, indicating the groundwater is unsuitable for drinking purposes.

Moreover, the long-term intake of groundwater containing excessive pollutants poses serious risks to human health. Reports on health issues arising from the consumption of groundwater exceeding standards for fluoride, arsenic, nitrate, and other pollutants are widespread worldwide [21–24]. Literatures have indicated that directly comparing analyzed contaminant levels with guideline limits is insufficient for assessing comprehensive hazard levels and identifying the most significant contaminants [25]. Health risk assessment, which emerged in the 1980s [26], quantitatively links environmental pollution to human health by evaluating the risks pollutants pose [27, 28]. This method provides crucial scientific support for risk management and policy-making, analyzing potential health impacts across various dietary and environmental sources [29]. By objectively estimating the degree and likelihood of harm, health risk assessment connects groundwater pollution with human health, aiding in health risk management, drinking water safety, and environmental protection [28, 30]. Jaiswal et al [27] assessed heavy metal levels in the Yamuna River during monsoon and non-monsoon seasons in Uttar Pradesh, India, identified possible contamination sources, and evaluated associated health risks, finding that the hazard index (HI) and fuzzy-logic hazard index (FHI) values for children exceeded safe limits at most sites during non-monsoon seasons and at a few sites during the monsoon season, while for adults, HI and FHI values remained within safe limits.

The Wulong River, the largest river in the Shandong Peninsula, consists of five major tributaries: Bailong River, Xian River, Qingshui River, Moshui River, and Fushui River. The main stream spans 52.5 km (from Wulongxia Gorge to Xiangdao) with a watershed area of 567.16 km^2^. The region is characterized by extensive fruit tree cultivation, and human activities over the years have significantly impacted groundwater quality, particularly regarding nitrate levels in groundwater [31]. As a crucial source of drinking water in the area, the lack of research on groundwater hydrochemistry and water quality poses certain risks to human health. In this study, we aim to (1) investigate the hydrogeochemical characteristics of groundwater, (2) identify the formation mechanisms of the hydrogeochemistry, (3) evaluate groundwater quality and; (4) assess the risk posed by nitrate in groundwater to human health through consumption of water. The results of the present study can provide a useful reference for the management and protection of groundwater.

## 2. Material and methods

### 2.1 Study area

#### 2.1.1 Location and climate

**Fig 1:**
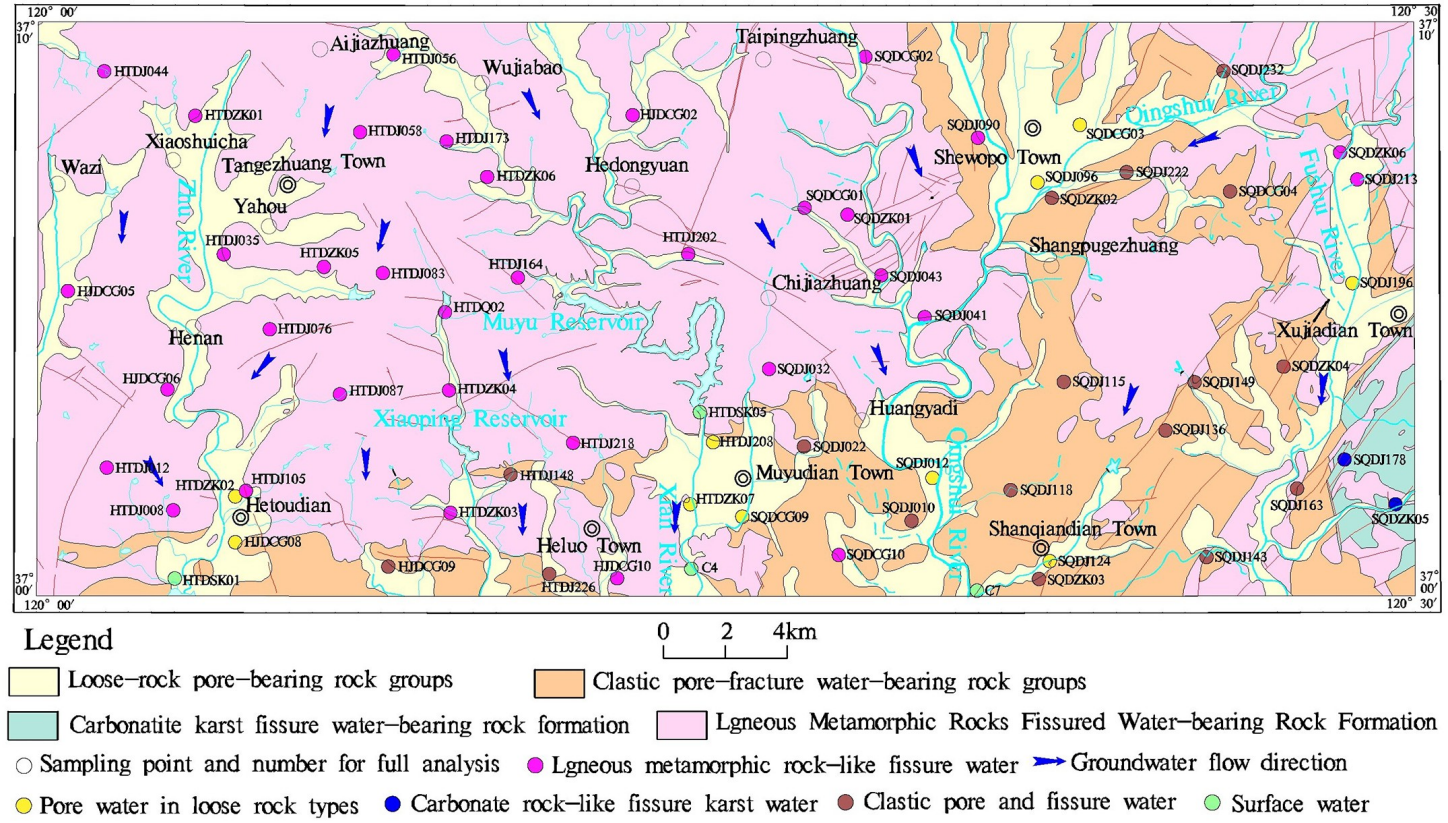
Location map of the study area with distribution of sampling points. The reivers and towns shown in the map was generated from USGS/NASA Landsat (https://landsat.visibleearth.nasa.gov/).

The study area is situated in the southwestern part of Yantai City, within the upper reaches of the Wulong River Basin. Its coordinates range from 120°30′E to 121°00′E and 37°00′N to 37°10′N, encompassing an approximate area of 840 km^2^ (Fig 1). The region is located in the eastern Shandong low mountain and hilly area, characterized by higher terrain in the north, lower terrain in the south, higher elevation in the east, and lower elevation in the west. The study area experiences a warm temperate East Asian continental monsoon climate, marked by distinct transitions between the four seasons. The average annual temperature is 11.3°C, with an average lowest temperature of -3.8°C and a highest of 24.6°C. The average annual rainfall amounts to about 650 mm. The surface water system in the study area is well-developed, constituting part of the upper reaches of the Wulong River Basin. Major rivers, such as the Xuan River, Qingshui River, and Fushui River, flow from north to south, eventually converging into the Wulong River and ultimately draining into the Yellow Sea.

#### 2.1.2 Formation and lithology

The study area is located at the junction of the North China Block, the Jiaoliao Uplift Zone, the southern margin of the Jiaobei Uplift Zone, and the northern margin of the Jiaolai Depression. It spans two major tectonic units, and the bedrock formations belong to the Jiaobei Formation in the Lüdong Stratigraphic Subdivision of the Jizheng-Lu Region of the North China Stratigraphic Region. The exposed formations in the area mainly include the Middle Proterozoic Tangjiazhuang Formation, the Neo-Proterozoic Jiaodong Formation, the Paleoproterozoic Jingshan Formation, the Fenzishan Formation, the Mesozoic Cretaceous Laiyang Formation, Qingshan Formation, Wangshi Formation, and the Neogene Quaternary. Ductile shear zones and northeast and northeast-southeast trending fault structures are well-developed, and volcanic rocks are distributed in a sheet-like pattern in the northwest, central, and northeast parts.

#### 2.1.3 Hydrogeological setting

According to the aquifer media, hydraulic properties, the study area can be divided into four major aquifer groups [31, 32]. (1) Pore aquifer of loose rocks, is distributed in the valley areas and the mountainous valleys upstream, characterized by mainly fine sand, medium-coarse sand, coarse sand, and gravelly sandstone. (2) Pore-fissure aquifer of clastic rocks, is found in the southern and eastern parts of the study area, with rock types including conglomerate, gravel-containing coarse sandstone, sandstone, fine sandstone, and shale. (3) Fractured karst aquifer of carbonate rocks, is located in the southeastern region, dominated by rocks such as fangshe marble and dolomite marble. (4) fractured aquifer of volcanic rocks-metamorphic rocks, is widely distributed in the study area, composed of magmatic and metamorphic rocks from the Middle Archean to the Middle Mesozoic Cretaceous. Groundwater in the region is recharged by atmospheric precipitation, and the overall flow direction is generally consistent with the topographic slope, flowing from north to south.

### 2.2 Data collection, sampling and analysis

In this study a total of 63 groups of groundwater samples (Fig 1) from public supply wells were collected in the study area in Jun 2020. Based on the hydrogeological setting, groundwater samples were classified into four types according to the aquifers from which they were taken: pore-fissure groundwater, pore groundwater, karst groundwater, and fissure groundwater. Prior to sampling, three well volumes of water were purged from the well and used to flush the equipment used to collect the sample. Immediately after collection, pH and TDS were measured with a multi-parameter portable meter (HACH HQ40D, USA). All the samples were taken directly at the wellhead. Water samples, carrying out chemical analyses of main components (K^+^, Na^+^, Ca^2+^, Mg^2+^, Cl^−^, SO_4_^2−^, HCO_3_^−^ and NO_3_^−^) were collected and stored in pre-cleaned 500 mL polyethylene bottles. Mg^2+^, Ca^2+^, and Na^+^ were determined by inductively coupled plasma optical emission spectroscopy (ICP-OES, PerkinElmer, Avio 200); Cl^−^ and SO_4_^2−^ were determined by ion chromatography (ICS-1100, Thermo Fisher Scientific, USA); HCO_3_^−^ was measured by acid-base titration according to APHA [33], and the total analysis error was less than 5%.

### 2.3 Statistical and hydrogeochemical analysis

#### 2.3.1 Correlation analysis

To identify the relationship among the various hydrogeochemical compositions, correlation analysis was carried out. Since most of the components do not satisfy the standard normal distribution, the Spearman R correlation was calculated using a predetermined significance level of 0.05 (5%).

#### 2.3.2 Principal component analysis (PCA)

To identify hydrogeochemical characteristics that were dominant for most of the dataset variability [15], principal component analysis (PCA) was performed. PCA is a statistical procedure that utilizes an orthogonal transformation to convert a set of possibly correlated variables/observations into a set of values of linearly irrelevant variables called principal components (or factors), and each factor is independent [4]. Varimax rotation and Kaiser normalization were used to identify the factor eigenvalue and limit the factor number. The factors with relatively large eigenvalues are associated with features that are most important in depicting the system variability.

#### 2.3.3 Water quality index (WQI)

The WQI is an effective tool for appraising the overall quality of groundwater, and is calculated as [30, 34]:

$$W_{i}=\frac{w_{i}}{\sum w_{i}}$$ (1)

$$WQI=\sum (W_{i}\times (\frac{C_{i}}{S_{i}})\times 100)$$ (2)

$${EW}_{i}=\frac{W_{i}\times (\frac{C_{i}}{S_{i}})}{WQI}\times 100$$ (3)

where *i* represents the sample number; *W*_*i*_ and *w*_*i*_ are the relative weight and weight of each index, respectively, as referenced from Şener et al [34] and Ravindra et al [12] (Table 1); *C*_*i*_ and *S*_*i*_ are the measured concentration and permissible value of each index, respectively; and *EW*_*i*_ is the effective weight of each index. WQI values can be categorized as non-drinkable (WQI ≥ 300), very poor (200 ≤ WQI < 300), poor (100 ≤ WQI < 200), good (50 ≤ WQI < 100), and excellent (WQI < 50).

**Table 1:** Relative weight of hydrochemical parameters [12, 34].

Parameters	WHO standards (2017) [35]	Weight (w_i_)	Relative weight (W_i_)
Na	200	2	0.0625
K	12	2	0.0625
Ca	300	2	0.0625
Mg	30	2	0.0625
Cl	250	3	0.09375
SO_4_	250	4	0.125
HCO_3_	300	3	0.09375
NO_3_	50	5	0.15625
TDS	500	5	0.15625
PH	8.5	4	0.125
		∑ w_i_ = 32	∑ W_i_ = 1

#### 2.3.4 Human health risk assessment

The assessment of the risk posed by groundwater quality to human health included four steps [7, 30]: (1) hazard identification; (2) dose effect analysis; (3) exposure assessment and; (4) risk characterization. Nitrate in groundwater is a non-carcinogenic toxic substance. Since the assessment of risk posed by a toxic substance is generally based on the reference dose, the non-carcinogen risk assessment model was used in the present study [34, 36, 37]:

$$HQ=\frac{ICD}{DRF}$$ (4)

In Eq (4) HQ is the non-carcinogenic risk index, ICD represents the average daily exposure dose of a non-carcinogenic substance(mg·kg^−1^·d^−1^), and DRF represents the reference dose of the noncarcinogenic substance (mg·kg^−1^·d^−1^). The reference dose for assessing the risk of nitrate to human health via consumption of potable water is 1.6 mg·kg^−1^·d^−1^. According to the USEPA health risk assessment guidelines [26], the threshold of non-carcinogenic risk HQ is 1. Therefore, a HQ < 1 indicates that the risk posed by a non-carcinogenic substance to human health is within an acceptable level. In contrast, HQ > 1 indicates that the risk posed by the non-carcinogenic substance to human health has reached an unacceptable level, and the value of HQ is positively related to the non-carcinogenic health risk [37, 38].

Nitrate in groundwater mainly enters the human body through consumption of potable water. The formula for calculating the average daily exposure dose (ICD) is [7, 30]:

$$ICD=\frac{C\times IR\times EF\times ED}{BW\times AT}$$ (5)

In Eq (5), C is the measured concentration of nitrate in groundwater (mg·L^−1^), IR is the drinking rate (L·d^−1^), EF is the exposure frequency (d·a^−1^), ED is the exposure duration (a), BW is the average body weight of residents (kg) and AT is life expectancy(d) [39]. Table 2 shows the parameters of the groundwater nitrate health risk assessment model used in the present study.

**Table 2:** Parameters of health risk assessment model used in the present study to assess the risk posed by nitrate in the groundwater to human health.

Parameters	Children	Adults
DRF (mg·kg^−1^·d^−1^)	1.6	1.6
IR (L·d^−1^)	1.5	2
BW (kg)	30	65
AT (a)	365×ED	365×ED
ED (a)	12	30
EF (d·a^−1^)	365	365

## 3. Results and discussion

### 3.1 Characteristics of groundwater hydrochemistry

#### 3.1.1 Groundwater hydrochemical components

The results of mathematical statistical analysis can reflect the basic status of groundwater chemical components in the work area over the recent period. Therefore, statistical characteristic value analysis was conducted on the groundwater hydrochemical parameters (Table 3).

**Table 3:** Statistical indicators of groundwater chemical components in study area.

Statistical indicators	Hydrochemisty components (mg/L)							pH	TDS (mg/L)
	Na^+^	Ca^2+^	Mg^2+^	Cl^−^	SO_4_^2−^	HCO_3_^−^	NO_3_^−^		
Maximum	227.00	266.00	63.60	164.00	257.00	720.9	593.00	8.4	1400.0
Minimum	8.04	5.24	2.05	2.10	5.89	23.6	0.22	7.3	77.4
Average	51.69	109.50	29.14	60.54	99.36	207.1	172.40	7.7	639.5
Standard deviation	39.79	52.16	13.97	32.03	54.33	122.4	144.15	0.2	279.5
Coefficient of variation (%)	76.98	47.63	47.94	52.91	54.68	59.1	83.59	3.0	43.7

Table 3 showed that Ca^2+^ is the predominant cation, followed by Na^+^ and Mg^2+^, with average ion concentrations of 109.50 mg/L, 51.69 mg/L, and 29.14 mg/L, respectively. The variation range of Na^+^ concentration among cations is 8.04 to 227 mg/L, with a coefficient of variation of 76.98%, making it the highest among cations. On the other hand, the variation range of Ca^2+^ concentration is 5.24 to 266 mg/L, with a coefficient of variation of 47.63%, making it the lowest among cations.

The concentration of Mg^2+^ ranged from 2.05 to 63.60 mg/L, with a coefficient of variation of 47.94%, similar to that of Ca^2+^.

HCO_3_^−^ is the predominant anion, followed by NO_3_^−^, SO_4_^2−^, and Cl^−^, with average ion concentrations of 207.10 mg/L, 172.40 mg/L, 99.36 mg/L, and 60.54 mg/L, respectively (Table 3). The variation range of HCO_3_^−^ concentration is 23.64 to 720.92 mg/L, with a coefficient of variation of 59.12%, making it the highest among anions. The concentration ranges for SO_4_^2−^ and Cl^−^ are 5.89–257.00 mg/L and 2.10–164.00 mg/L, respectively, with their variation coefficients being relatively similar at 54.68% and 52.91%. The variation coefficients of each ion are all above 50%, indicating significant variability in the hydrochemical characteristics of local groundwater. The coefficients of variation for hydrochemical parameters are relatively high, indicating that the aquifers in the study area are fragmented and not part of a single aquifer system [31]. This suggests limited connectivity between groundwater in different regions, consistent with the lack of a unified groundwater flow direction shown in Fig 1, indicating poor connectivity between groundwater sources [31, 32].

NO_3_^−^ exhibits significant spatial variability, with a variation range of 0.22 to 593 mg/L and a coefficient of variation of 83.59%, making it the highest among all ions. The significant variability in NO_3_^−^ concentrations, primarily originating from agricultural fertilizer pollution [13, 21], indicates that NO_3_^−^ concentration changes are influenced by local agricultural activities.

Overall, the complex hydrogeological conditions in the study area, coupled with the fragmented nature of the aquifer system and varying intensities of human activities across different regions, have resulted in significant variations in the concentrations of anions and cations in the groundwater.

#### 3.1.2 Groundwater types

**Fig 2:**
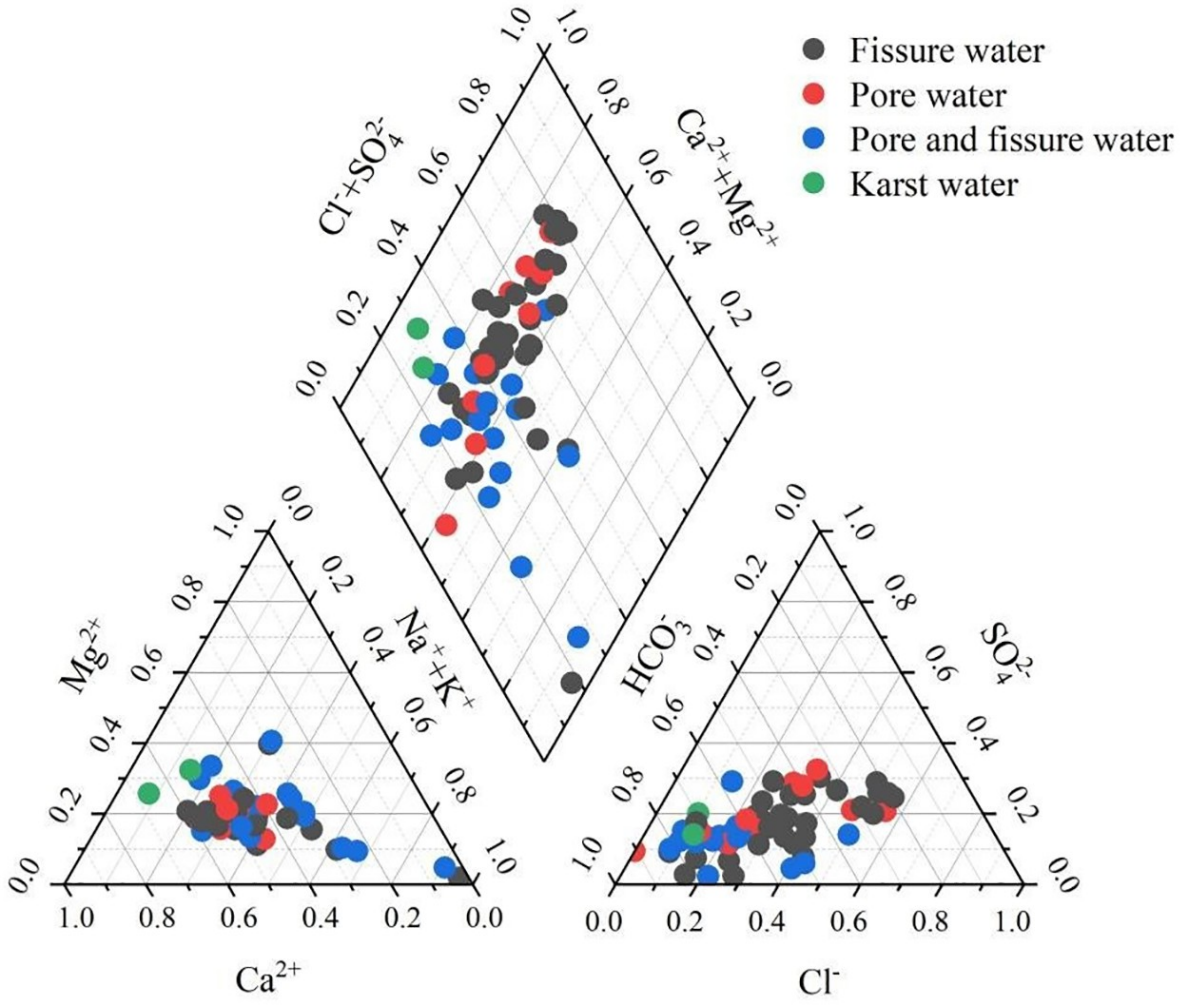
Piper diagram of groundwater in study area.

The Piper trilinear diagram not only quantifies the proportional content of different ions but also reveals the evolutionary patterns of groundwater chemical components [40]. From the groundwater Piper diagram in the study area (Fig 2), the predominant cations are Ca^2+^ and Mg^2+^, while the predominant anions are HCO_3_^−^ and SO_4_^2−^.

**Fig 3:**
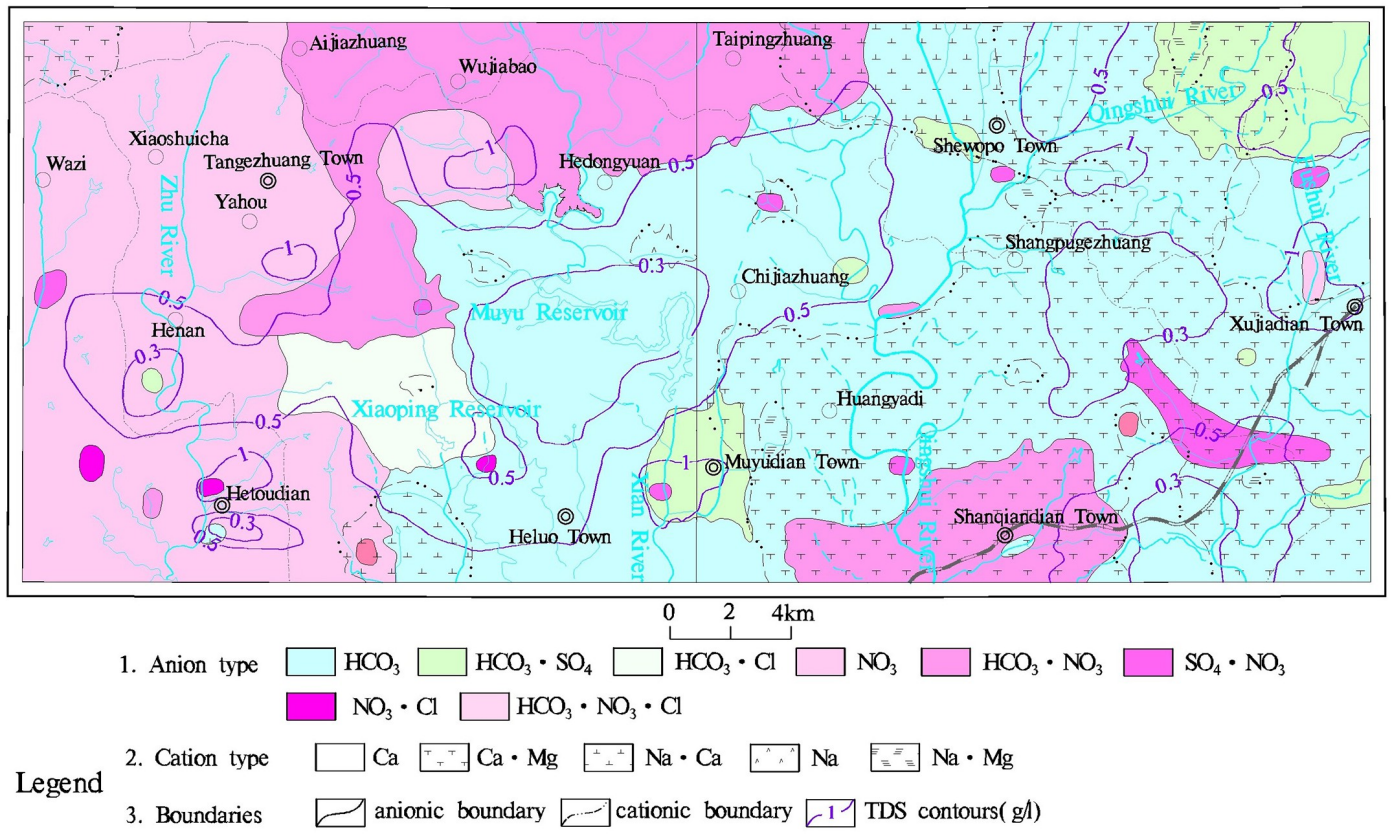
Groundwater hydrochemical types distribution in study area. The reivers and towns shown in the map was generated from USGS/NASA Landsat (https://landsat.visibleearth.nasa.gov/).

Influenced by factors such as topography, lithology, geological structures, and human activities, different types of groundwater in the study area exhibit distinct chemical characteristics (Fig 3). The chemical types of pore water mainly include HCO_3_·SO_4_-Ca and HCO_3_-Ca types. The karst water is primarily of the HCO_3_-Ca type. The chemical types of pore-fracture water in clastic rocks are dominated by HCO_3_-Ca and HCO_3_-Mg types, with localized distribution of HCO_3_-Na type. The chemical types of fracture water in igneous and metamorphic rocks are mainly HCO_3_·SO_4_-Ca and HCO_3_-Ca types. As a result, the classification of hydrochemical types in the study area is quite diverse, which is consistent with the significant variations observed in the groundwater hydrochemical parameters.

In this study area, NO_3_^−^ concentration is relatively high, but in the traditional Shukalev classification, it does not reflect the proportion of NO_3_^−^. Therefore, this paper incorporates NO_3_^−^ as an integral part of the anions in the Shukalev classification method to re-analyze the hydrochemical types of groundwater in this area (Fig 3). The predominant cation types in the study area are primarily Ca and Ca·Mg, while the most significant anion is HCO_3_^−^, followed by NO_3_^−^, SO_4_^2−^ and Cl^−^. This is consistent with the results of the Piper diagram.

In the study area, Ca, Mg and HCO_3_^−^ type water is mainly found in the eastern region, where sandstone and shale layers are present. This region experiences less pollution due to the weak weathering of the rock layers, with a weathering thickness of 1 to 10 m [32], making surface pollutants less likely to infiltrate groundwater.

NO_3_^−^ type water is widely distributed in the study area, mainly in the western, northwestern, and southeastern parts of the study area. This distribution is closely related to local agricultural cultivation, indicating significant pollution from agricultural activities [19]. Long-term consumption of groundwater with high nitrate concentrations may lead to elevated levels of methemoglobin, carcinogenesis in the digestive system, and other health issues. Moreover, NO_3_^−^ type water is predominantly distributed in areas with magmatic and metamorphic rocks. These rocks, influenced by weathering and tectonic activities, develop network-like weathering fractures and linear structural fractures. Due to differences in formation periods and weathering resistance of various lithologies, the weathering layer in this region is about 20 to 35 meters thick [32]. The weathering of the surface layer facilitates the infiltration of surface pollutants, leading to groundwater contamination. This is the reason for conducting a human health risk assessment in the present study.

### 3.2 Formation mechanisms of the hydrogeochemistry

#### 3.2.1 Correlation of hydrochemical components

Correlation analysis can be used to analyze multiple sets of variable elements, reflecting the degree of correlation between ions. The closer |R| is to 1, the stronger the significance and the greater the correlation [41]. Table 4 presents the results of the correlation analysis of eight chemical components.

**Table 4:** Correlation coefficient of chemical composition of groundwater.

	Na^++K+^	Ca^2+^	Mg^2+^	Cl^−^	SO_4_^2−^	HCO_3_^−^	NO_3_^−^	TDS
Na^++K+^	1							
Ca^2+^	0.415**	1						
Mg^2+^	0.420**	0.714**	1					
Cl^−^	0.562**	0.663**	0.479**	1				
SO_4_^2−^	0.450**	0.784**	0.700**	0.419**	1			
HCO_3_^−^	0.388**	0.081	0.424**	-0.048	0.164	1		
NO_3_^−^	0.388**	0.782**	0.508**	0.643**	0.555**	-0.288*	1	
TDS	0.675**	0.895**	0.757**	0.679**	0.812**	0.162	0.825**	1

*Correlation is significant at the 0.05 level (2-tailed).

**Correlation is significant at the 0.01 level (2-tailed).

Significant values are in bold.

It can be observed that there is a strong positive correlation (R^2^>0.65) between TDS and Na^++K+,^ Ca^2+^, Mg^2+^, Cl^−^, SO_4_^2−^, NO_3_^−^ indicating that solutes in the groundwater mainly originate from mineral dissolution and anthropogenic pollution [4, 42]. There is a positive correlation between Na^++K+^ and Cl^−^, but the correlation is not strong (R^2^>0.562), suggesting sources for Na^++K+^ or Cl^−^ beyond halite. Strong correlations between Ca^2+^ and Mg^2+^, Cl^−^, SO_4_^2−^ imply the influence of sulfate and silicate mineral dissolution. There is a strong correlation between NO_3_^−^ and TDS, Ca^2+^, Cl^−^ indicating that human activities have impacted the natural groundwater chemical formation mechanism, specifically affecting the concentrations of Ca^2+^, Mg^2+^ and SO_4_^2−^ in groundwater. The strong positive correlation (R^2^ = 0.679) between NO_3_^−^ and Cl^−^ suggests that Cl^−^ also originates from human pollution [43].

#### 3.2.2 Gibbs diagram

The Gibbs diagram model provides a clear representation of the natural sources and chemical formation mechanisms that control groundwater chemistry [18, 44, 45]. These mechanisms include precipitation, rock weathering, and evaporation-concentration [44].

**Fig 4:**
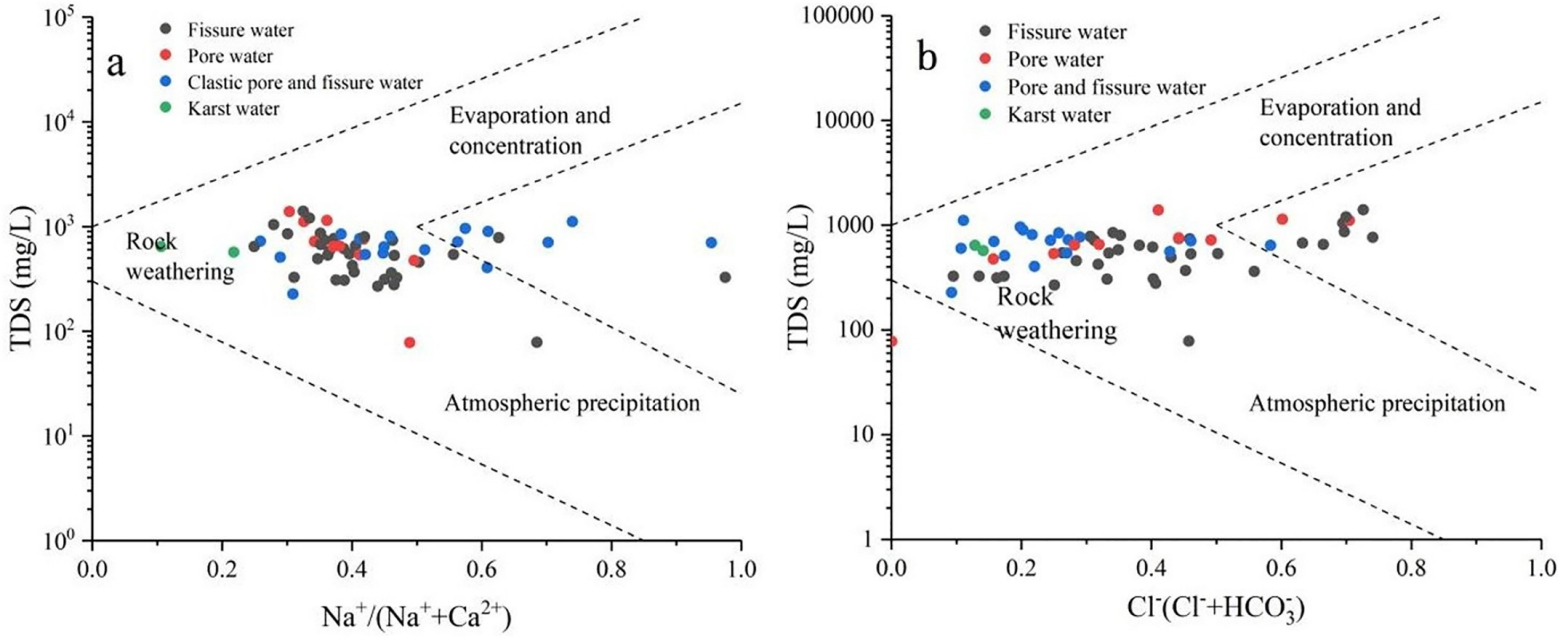
Gibbs model of groundwater chemistry.

Analyzing the groundwater chemistry Gibbs diagram in the study area (Fig 4), it is indicated that TDS content mostly ranges from 200 to 1400 mg/L. The Na^+^/(Na^+^+Ca^2+^) ratio is primarily between 0.2 and 0.68, while the Cl^−^/(Cl^−^+HCO_3_^−^) ratio is mainly between 0.1 and 0.74. Most sampling points are located near areas influenced by rock weathering and away from regions controlled by atmospheric precipitation. This suggests that the ion composition of groundwater in the basin is mainly controlled by the weathering of rock minerals. The groundwater flow conditions are favorable, and water-rock interaction is substantial, while the impact of precipitation and evaporation-crystallization is noticeably weaker. Some sampling points are situated in the middle-right section of the Gibbs diagram, with TDS content mostly ranging from 700 to 1100 mg/L and Na^+^/(Na^+^+Ca^2+^) ratios between 0.7 and 1.0. This indicates that the groundwater around these sampling points may be influenced by external Na^+^ inputs due to anthropogenic pollution or cation exchange [46].

#### 3.2.3 Ion ratios

**Fig 5:**
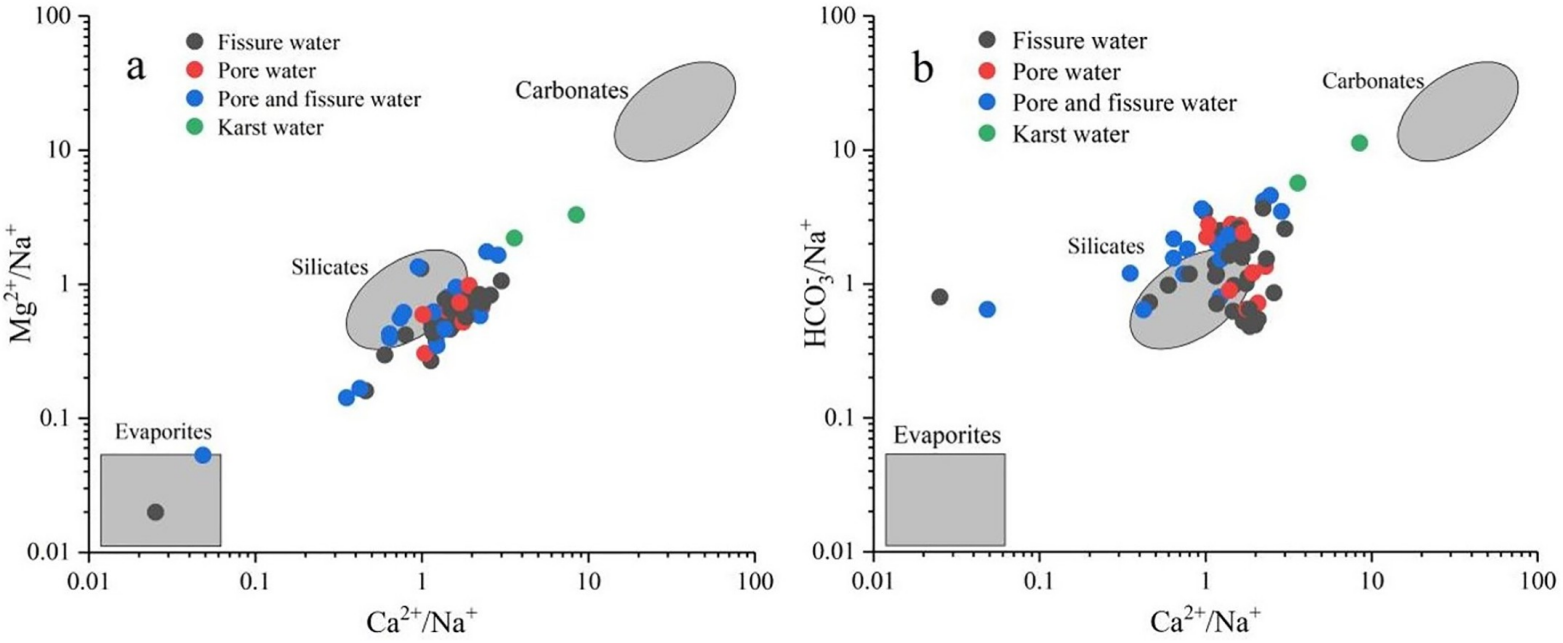
End-member diagram of groundwater.

Groundwater ion ratio analysis can be employed to study the sources of major ions in water and the evolution of hydrochemistry [4, 12, 47]. From Fig 5, it can be documented that the groundwater sampling points in the study area are mainly distributed between silicate and carbonate mineral end-members. This indicates that silicate and carbonate minerals play a dominant role in the weathering processes of groundwater in this region, with a weak influence from evaporite dissolution. Considering the geological conditions within the area, it is found that the northern and central low hills are primarily composed of silicate minerals such as granite and diorite. In contrast, the eastern and southeastern parts are mainly characterized by carbonate minerals like the Jing Shan Group dolomite. After undergoing weathering and leaching processes, these minerals contribute to the hydrochemical system as groundwater flows into the area.

**Fig 6:**
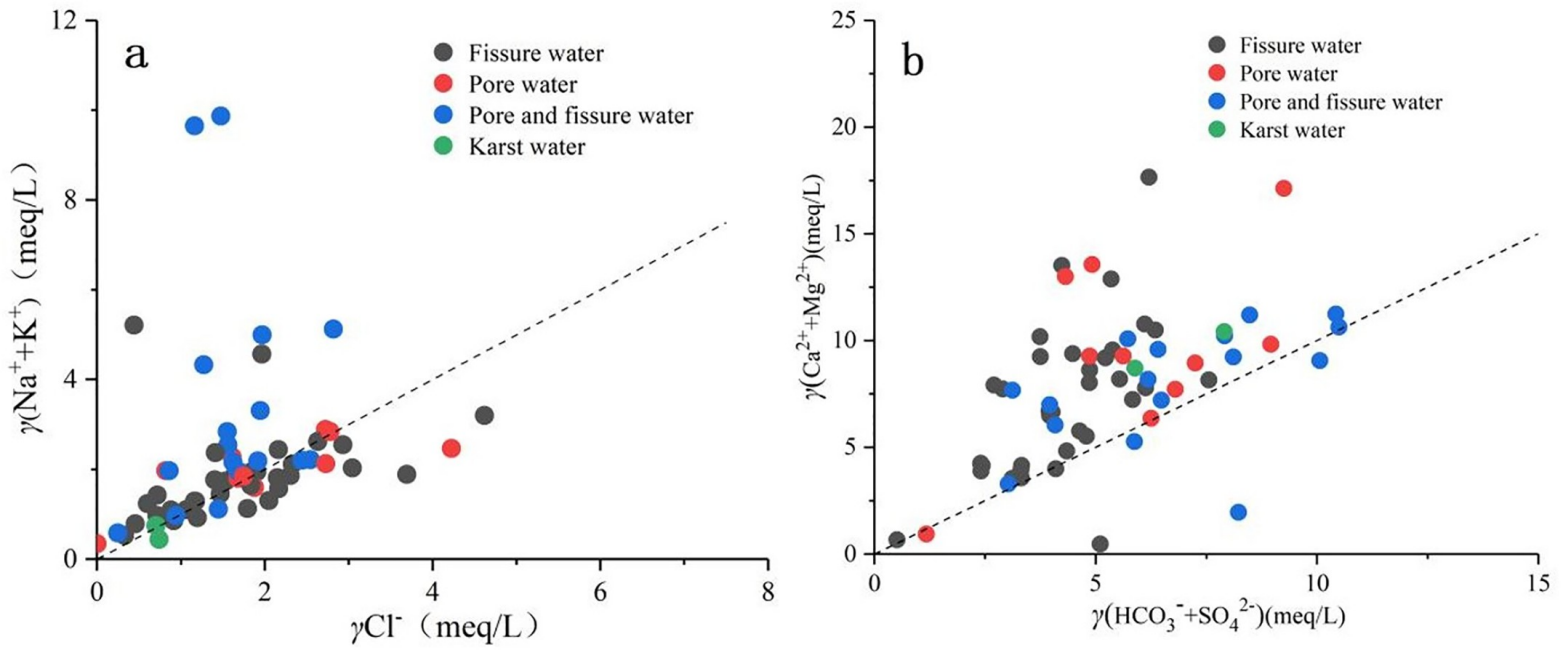
Groundwater ion ratio of different types groundwater in study area.

The milliequivalent ratio of major ions in groundwater can, to some extent, reflect the impact of different rock weathering on the sources of major chemical components and the hydrochemical genesis. When the γ(Na^+^+K^+^)/γCl^−^ value in groundwater approaches 1, it indicates that Na^+^ and K^+^ primarily originate from the dissolution of halite. From Fig 6(a), it is suggested that most groundwater sampling points in the study area are located above the 1:1 line, suggesting that Na^+^ and K^+^ ions in groundwater result from the combined effects of halite dissolution and leaching of silicate minerals. Additionally, a small number of water samples below the 1:1 line suggests possible influences from cation exchange, agricultural pollution or increased chloride content from domestic wastewater.

The ratio of γ(Ca^2+^+Mg^2+^) and γ(HCO_3_^−^+SO_4_^2−^) can be used to analyze the sources of Ca^2+^ and Mg^2+^ in groundwater. When the γ(Ca^2+^+Mg^2+^)/γ(HCO_3_^−^+SO_4_^2−^) value is close to 1, it indicates that Ca^2+^ and Mg^2+^ in groundwater primarily originate from the dissolution of evaporite or calcium-magnesium silicate minerals [48]. Fig 6(b) reveals that the majority of groundwater sampling points in the study area are located above the 1:1 line, indicating that Ca^2+^ and Mg^2+^ in groundwater mainly originate from the dissolution of calcium-magnesium silicate minerals, not from sulfate and carbonate minerals. In Table 4, the correlation coefficients for Cl^−^, NO_3_^−^, and Ca^2+^ are 0.77 and 0.85, respectively, indicating a strong positive correlation. This suggests that human activities impact the dissolution of calcium-bearing minerals. Ma et al [49] has indicated that exogenous acids generated by human activities, such as sulfuric acid or nitric acid, can lead to the release of Ca^2+^ in groundwater. This process reduces the amount of carbonate reaction, consequently decreasing the concentration of HCO_3_^−^ in groundwater.

#### 3.2.4 Cation exchange

**Fig 7:**
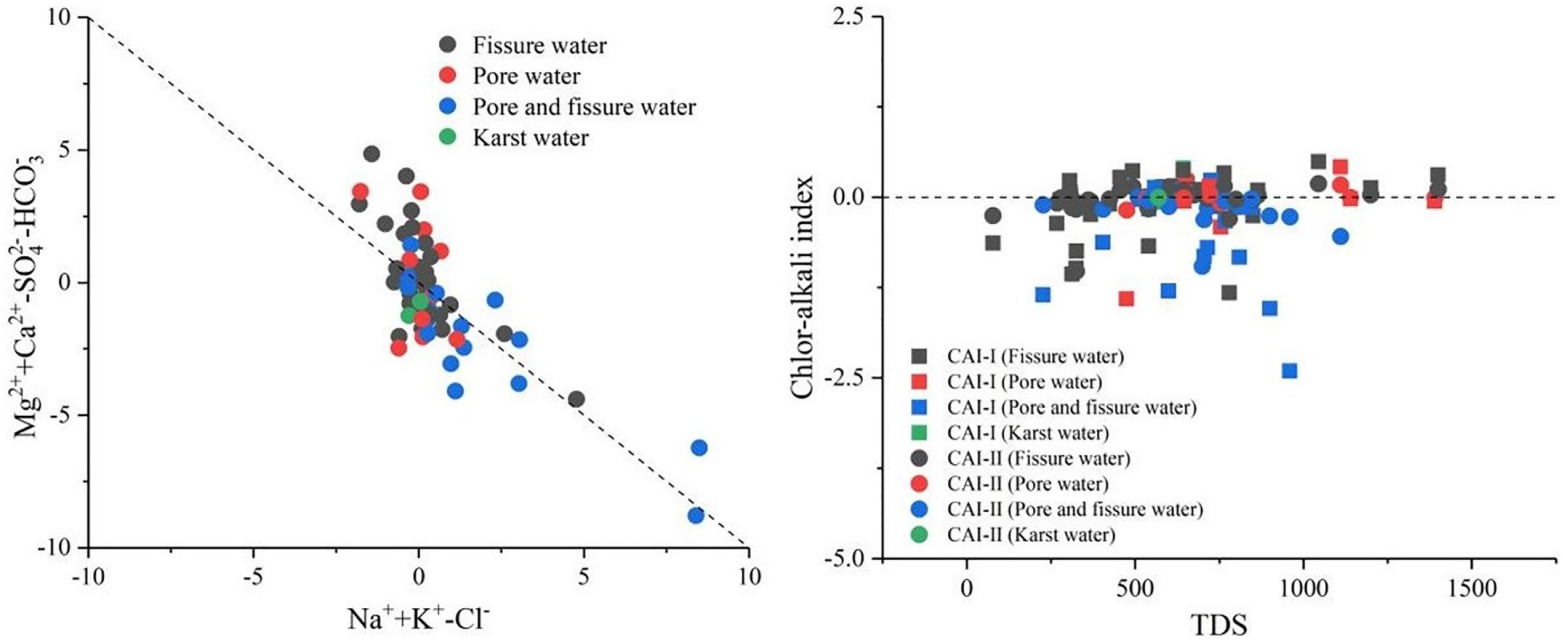
Cation exchange and adsorption of groundwater and chlor-alkali index of groundwater.

The cation exchange adsorption is a phenomenon where, under certain conditions, particles adsorb certain ions from water, converting some of the originally adsorbed cations into components of the water [45, 50]. The relationship diagram of γ(Na^−^+K^+^-Cl^−^) and γ[(Ca^2+^+Mg^2+^)-(SO_4_^2−^+HCO_3_^−^)] can reflect the cation exchange in groundwater. From Fig 7(a), it can be observed that groundwater sampling points in the study area are distributed along a line with a slope of -0.4, with an R^2^ value of 0.496. Most samples are around the ratio line with a slope of -1, indicating the presence of cation exchange in groundwater [43].

To further analyze whether the cation exchange in groundwater is a forward or reverse reaction, the chloride-alkalinity index, also known as the Schoeller index [51, 52], is used. The formulas for calculating Schoeller indices CAI-I and CAI-II are as follows (ion concentration unit: meq·L^-1^):

$$CAI­I=\frac{{Cl}^{-}-({Na}^{+}+K^{+})}{{Cl}^{-}}$$ (6)

$$CAI­II=\frac{{Cl}^{-}-({Na}^{+}+K^{+})}{{{HCO}_{3}}^{-}+{{SO}_{4}}^{2-}+{{CO}_{3}}^{-}+{{NO}_{3}}^{-}}$$ (7)

If the Schoeller index is a negative value, it indicates that Na^+^ and K^+^ in the aquifer’s lithogenic minerals are replaced by Ca^2+^ and Mg^2+^ from the water, with Ca^2+^ and Mg^2+^ precipitating and adhering to the minerals. Conversely, if a positive value is obtained, it signifies the occurrence of the opposite cation exchange, where Ca^2+^ and Mg^2+^ in the lithogenic minerals of the aquifer are replaced by Na^+^ and K^+^ [52]. In Fig 7(b), the majority of the chloride-alkalinity indices are negative, indicating a positive ion exchange in the groundwater. This means that Na^+^ and K^+^ from the lithogenic minerals in the aquifer are exchanged with Ca^2+^ and Mg^2+^ in the water, leading to a decrease in Ca^2+^ and Mg^2+^ and an increase in Na^+^ and K^+^ in the groundwater.

#### 3.2.5 Factor analysis

Using SPSS for factor analysis of the 8 hydrochemical parameters collected from the samples (Table 5), 2 principal factors were extracted based on the cumulative variance contribution rate, reaching 80.005%, which can reflect the basic information of the original hydrochemical data.

**Table 5:** Factor analysis of groundwater variables.

Parameters	F1	F2
Na+K	0.159	0.779
Ca	0.960	0.023
Mg	0.684	0.43
Cl	0.834	-0.051
SO_4_	0.783	0.335
HCO_3_	-0.078	0.950
NO_3_	0.918	-0.195
TDS	0.943	0.303
Variability (%)	55.768%	24.238%
Cumulative %	55.768%	80.005%

F1 shows a variability of 55.768% with strong loadings for Ca, Mg, Cl, SO_4_, NO_3_, and TDS, respectively. The sources of Ca, Mg, and SO_4_ are attributed to the weathering and dissolution of carbonate and sulfate minerals. Cl and NO_3_ originate from the halite dissolution and influence of human pollution, as indicated by the analysis above. Therefore, F1 reflects both geogenic and anthropogenic sources of contamination in the study area.

F2 shows a variability of 24.238% with strong loadings for Na, K, and HCO_3_, respectively. Combining the analysis above, Na and K partially originate from the dissolution of halite and partially from cation exchange. HCO_3_ primarily comes from the weathering and dissolution of carbonate minerals. Hence, F2 reflects the geogenic source of groundwater hydrochemistry.

#### 3.2.6 Effects of human activity

**Fig 8:**
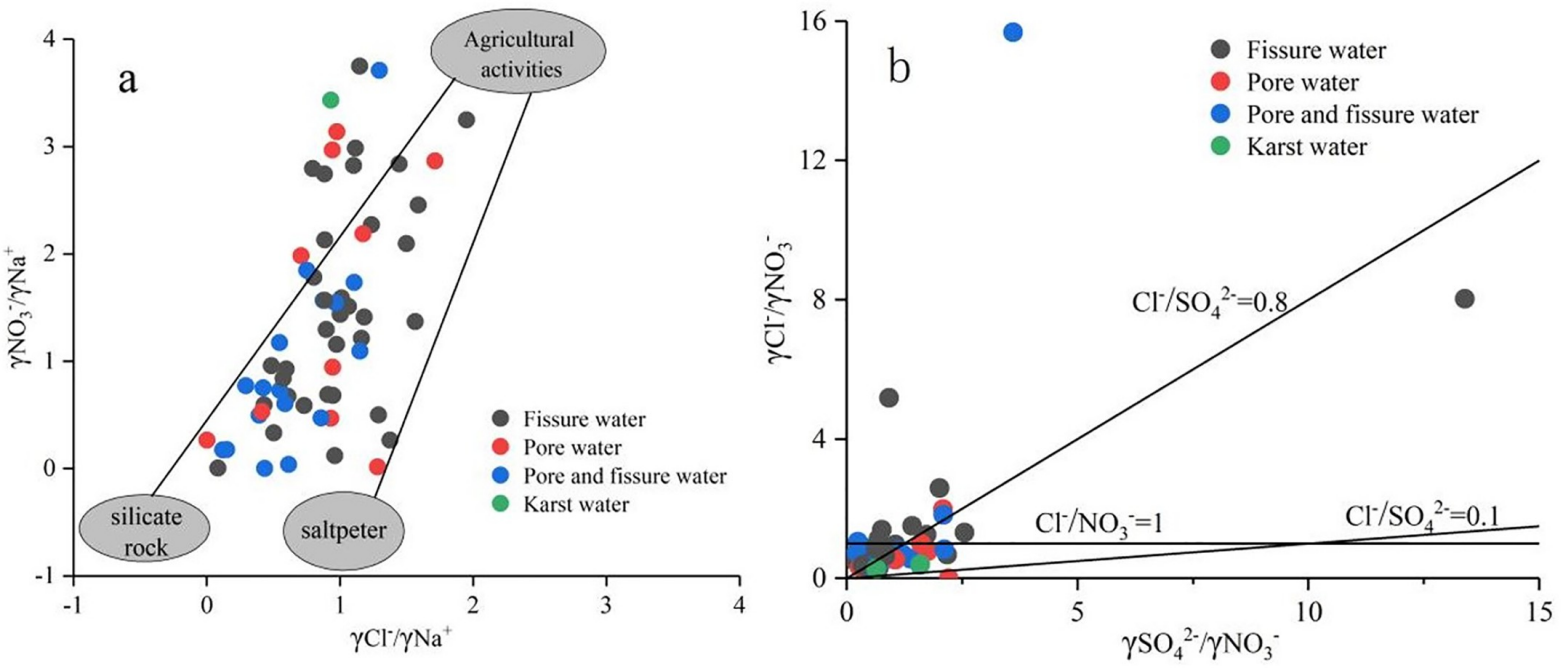
Relationship between the ratio of NO3^−^/Na^+^ and Cl^−^/Na^+^, Cl^−^/NO3^−^ and SO42^−^/NO3^−^ in groundwater.

The evolution of groundwater chemical composition is closely related to human activities, especially in areas with high human activity where concentrations of ions such as NO_3_^−^, Cl^−^ and SO_4_^2−^ are elevated. The higher the ratios of Cl^−^/Na^+^ and NO_3_^−^/Na^+^, the more pronounced the impact of human activities on groundwater [11]. As observed in Fig 8(a), some sampling points deviate from the silicate and rock salt end towards the agricultural activity end. This indicates a significant influence of human activities on groundwater, with NO_3_^−^ primarily originating from agricultural and livestock production activities such as fruit tree cultivation. Pollutants continuously migrate during the process of atmospheric precipitation and surface water infiltration, affecting the hydrochemical characteristics of local groundwater.

From Fig 8(b), it is known that sampling points are distributed more uniformly on both sides of the line where the Cl^−^/NO_3_^−^ ratio is 1, indicating the influence of residents’ activities on groundwater. The overall Cl^−^/SO_4_^2−^ ratio is relatively small, suggesting localized influence from industrial activities on groundwater. The ratios of SO_4_^2−^/NO_3_^−^ are also generally small, indicating that groundwater in the study area is additionally affected by agricultural pollution.

### 3.3 Water quality assessment

**Fig 9:**
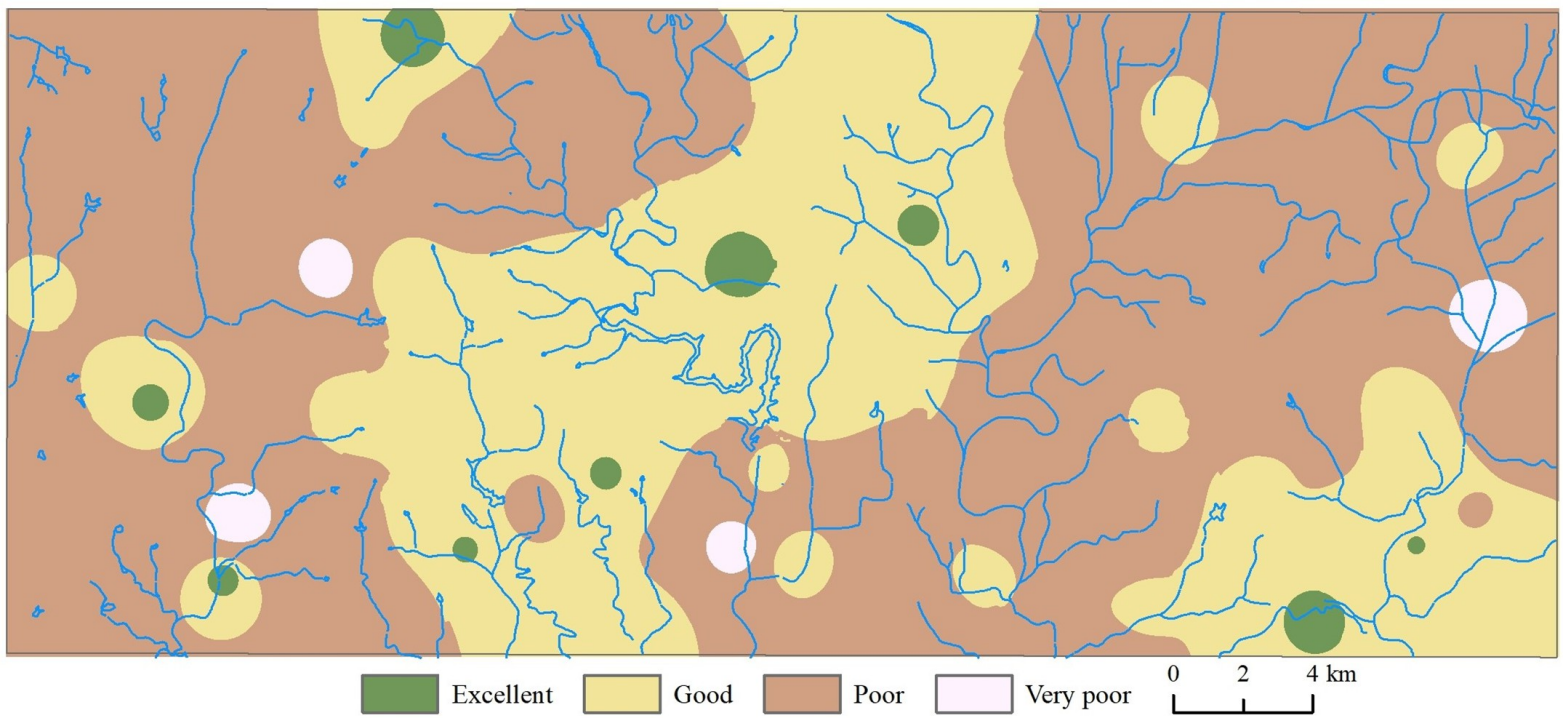
Spatial variation in groundwater quality within the central region of Shandong Province, China based on the water quality index (WQI). The reivers shown in the map was generated from USGS/NASA Landsat (https://landsat.visibleearth.nasa.gov/).

**Fig 10:**
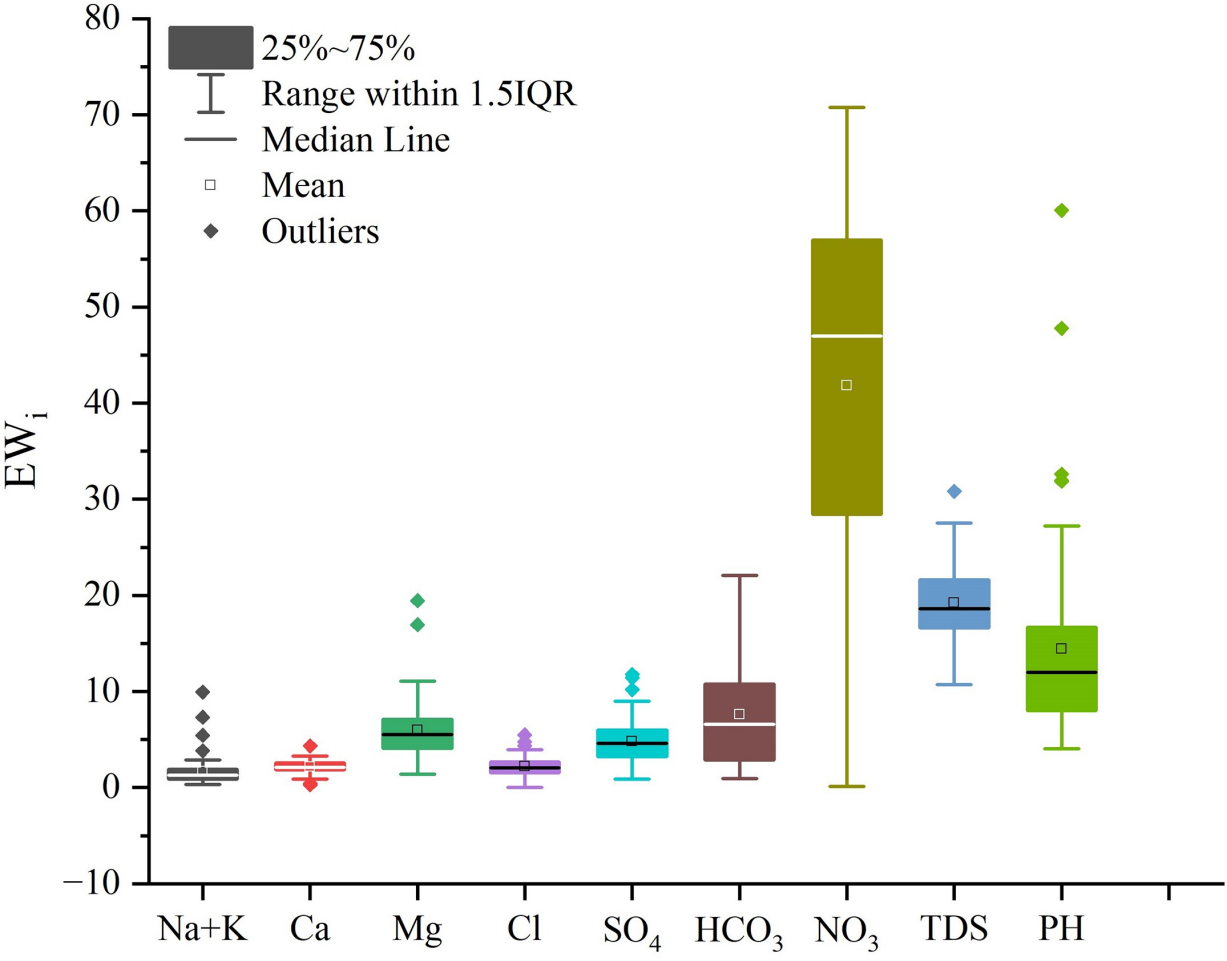
Histogram of each hydrochemical parameter effective weight.

Assessing groundwater quality is crucial for determining the safety of regional drinking water. In this study, no groundwater samples were classified as "Non-drinkable," with the majority falling into the "Poor" category (Fig 9). Groundwater quality tends to be better in the central regions and poorer in the surrounding areas (Fig 9). Specifically, 1.4% of groundwater was classified as "Excellent," 36.6% as "Good," 60.8% as "Poor," and 1.2% as "Very Poor." The average *Ew*_*i*_ for NO_3_^−^ is the highest, indicating that nitrate has the most significant impact on the WQI (Fig 10).

Regions with better groundwater quality are mostly found in less agriculturally developed areas, such as mountainous regions and groundwater recharge zones. These areas are less affected by agricultural pollution and are in groundwater recharge zones with fast groundwater flow rates and high renewal rates, contributing to relatively good water quality. On the other hand, areas with poorer water quality coincide with regions of higher nitrate concentration in groundwater, often found in agricultural planting areas like orchards. This indicates that agricultural activities are the primary source of pollution in the region.

Thus, the groundwater quality in the study area is influenced by a combination of natural factors and anthropogenic pollution, with agricultural pollution being a major contributor.

### 3.4 Human health risk assessment (HHRA)

HQ values from NO_3_^−^ exposure vary significantly in the study area. The HQ value for children in the study area ranges between 0.00688 to 18.53 with a mean value of 5.39; 82.54% of the samples have HQ values greater than 1. Meanwhile, for adults, the HQ values range from 0.00423 to 11.40 with an average value of 3.32; 79.37% of the samples have HQ values greater than 1. These findings demonstrated that drinking NO_3_^−^-contaminated water poses a significant health risk to most inhabitants in the study area. As a result, areas exposed to NO_3_^−^ should take considerable measures to protect inhabitants from NO_3_^−^ exposure.

In the Shandong Peninsula, there are many regions similar to our study area, mostly characterized by agriculture as the primary economic industry, particularly producing various agricultural products such as fruits and vegetables [53, 54]. These areas rely mainly on groundwater as their primary water source. However, the development of agriculture poses a threat to the quality of groundwater, leading to groundwater pollution. As indicated in this study, the issue of nitrate contamination is prominent, and the assessment of human health risks also suggests potential harm from nitrate contamination. Nevertheless, investigations and research on groundwater pollution issues in such regions are still relatively scarce. There is a need to intensify research efforts to establish a foundation for protecting human drinking water safety and overall health.

## 4. Conclusions

In this paper, we conducted a study on the hydrochemical formation mechanisms, quality and risk to human health of groundwater in the upper reaches of the Wulong River basin using mathematical analysis, Piper trilinear diagram, ion ratio method, and other approaches. The main conclusions are as follows:

1. The groundwater in the upper reaches of the Wulong River basin is generally neutral to weakly alkaline, with relatively concentrated distributions of cations and anions. The dominant cations are Ca^2+^ and Mg^2+^, while the main anions are HCO_3_^−^ and SO_4_^2−^. The hydrochemical types of groundwater mainly include HCO_3_·SO_4_-Ca, HCO_3_-Ca and HCO_3_-Na types. Due to the fragmented and discontinuous nature of the aquifers, the hydrochemical composition and types of groundwater in the region vary significantly. In the Wulong River basin, the hydrochemical characteristics of groundwater are influenced by the dissolution of silicate and carbonate minerals, cation exchange, and anthropogenic pollution, with nitrate pollution from human activities being the most prominent.
2. The groundwater in the study area does not have any samples classified as "Non-drinkable." The majority are categorized as "Good" (36.6%) and "Poor" (60.8%). Samples classified as "Very poor" and "Excellent" account for only 1.2% and 1.4%, respectively. Among the hydrochemical parameters, the *Ew*_*i*_ for NO_3_^−^ is the highest, indicating that the groundwater in the study area is severely contaminated by anthropogenic NO_3_^−^ pollution.
3. Human health risk assessment reveals that water samples posing exposure risks to children and adults account for 82.54% and 79.37%, respectively. There is a need to enhance research and protection efforts for groundwater in similar regions, such as the Shandong Peninsula.

## Supporting information

S1 Data (XLSX)
